# ASF1a inhibition induces p53-dependent growth arrest and senescence of cancer cells

**DOI:** 10.1038/s41419-019-1357-z

**Published:** 2019-01-28

**Authors:** Yujiao Wu, Xidan Li, Jingya Yu, Magnus Björkholm, Dawei Xu

**Affiliations:** 1 Division of Hematology, Department of Medicine, and Center for Molecular Medicine, Karolinska Institutet and Karolinska University Hospital Solna, Stockholm, Sweden; 2 Department of Medicine, Integrated Cardio Metabolic Centre (ICMC), Karolinska Institutet, Stockholm, Sweden

## Abstract

Anti-silencing function 1a (ASF1a) is a histone H3–H4 chaperone isoform involved in chromatin assembling and transcription regulation. Recently, ASF1a has been shown to be up-regulated in certain human malignancies and required for the expression of telomerase reverse transcriptase (TERT), a factor essential for the immortal phenotype of cancer cells; however, its role in oncogenesis remains poorly defined. In the present study, we determine whether ASF1a is required for the unlimited proliferation of cancer cells, a key cancer hallmark. Elevated ASF1a mRNA expression was observed in hepatocellular carcinoma (HCC) tumors. The overexpression of ASF1a was similarly found in 20 cancer types contained in TCGA and GTEx datasets. ASF1a knockdown led to growth arrest and senescence of wild-type (wt) p53-carrying HCC and prostate cancer cells. Cellular senescence mediated by ASF1a inhibition resulted from the robust up-regulation of p53 and p21^cip1^ expression, but without detectable changes in TERT expression. p53 inhibition attenuated p21^cip1^ induction caused by ASF1a depletion. Mechanistically, ASF1a-knocked down cells displayed widespread DNA damage. The TCGA dataset analysis revealed a negative correlation between ASF1a and p21^cip1^ expression in multiple types of primary tumors, including HCC, prostate, gastric, and breast cancer. Higher ASF1a and lower p21^cip1^ expression predicted a poor outcome in patients with HCC. Our results reveal that ASF1a overexpression is widespread in human malignancies and is required for the infinite proliferation of cancer cells, whereas its inhibition induces DNA damage and subsequent up-regulation of p53-p21^cip1^ expression, thereby triggering cellular senescence. Thus, ASF1a may serve as a potential target in cancer therapy.

## Introduction

Anti-silencing function 1 (ASF1), the most conserved histone H3–H4 chaperone, plays an important role in DNA replication, gene expression, DNA repair, and nucleosome assembly^1,2^. ASF1 is present as a single protein in yeast, while in the path of evolution, it duplicated to be two paralogs namely ASF1a and ASF1b^3^. ASF1a and ASF1b preserved most of their ancestors' conserved characters while they also developed novel and distinct functions. For example, ASF1a plays a crucial role in histone H3K56 acetylation and cellular reprogramming, whereas ASF1b is involved in proliferation regulation^1,4,5^. Recently, ASF1s have emerged as an oncogenic driver. ASF1b was shown to stimulate the proliferation of breast cancer cells and correlate with poor clinical outcomes^6^, whereas ASF1a promotes gastrointestinal cancer development and progression by activating β-catenin target genes^7^. Interestingly, ASF1a was reported to be required for the constitutive expression of telomerase reverse transcriptase (TERT), the telomerase catalytic component essential for the immortal phenotype of cancer cells^8^, which indicates that targeting ASF1a may reverse the unlimited proliferation of cancer cells via TERT inhibition.

Cellular senescence is a process in which cells exit the cell cycle and undergo distinctive phenotypic alterations, including morphology, chromatin, transcriptome, and secretome changes^9–12^. By limiting the replicative life span of somatic cells, senescence serves as a potent barrier to malignant transformation^13^. Under certain settings, cellular senescence could be more significant than cell death for tumor suppression, because subtle perturbations in senescence regulatory network influence cancer susceptibility dramatically in mice whereas defects in apoptosis do not^13^. Thus, cellular senescence induction has been suggested as a novel anti-cancer strategy.

There are several causes of cellular senescence, including persistent telomeric/genomic damage, too strong mitogenic signals, epigenomic perturbations, and oncogene activation^10^. Telomeres protect the ends of linear chromosomes and shorten with cellular proliferation^10^. A too short telomere increases genomic instability^9,10^, triggers DNA damage response (DDR), and thereby induces p53–p21^cip1^ and/or p16^ink4^–pRB pathway activation, ultimately leading to growth arrest and cellular senescence^9,10^. Oncogenes such as H-RAS can provoke senescence by super-stimulating the mitogen-activated protein kinase (MAPK) signaling^10^. Epigenetic changes like global chromatin relaxation have also been shown to promote senescence-associated heterochromatin formation by de-repressing the *CDKN2A* gene transcription^14^. Under certain conditions, epigenetic perturbations can trigger DDR without physical DNA damages^10^. Notably, no matter what the initiator is, most signals eventually activate the p53/p21^cip1^ and/or p16^ink4a^/pRB pathways through which senescence is induced^9,15,16^.

The renowned tumor suppressor p53 is considered as “the guardian of genome” by sensing and regulating the components of DDR, and promoting growth arrest and cellular senescence^17^. Once activated by upstream signals, p53 accumulates on the distal region of the p21^cip1^ (CDKN1A) promoter, transcriptionally enhancing p21^cip1^ expression. The p21^cip1^ protein inhibits several cyclin–CDK complexes and induces cell cycle arrest at the G1–S transition point, thereby serving as the final effector of growth arrest and cellular senescence. In HCC, the p53-p21^cip1^ signaling was reported as a crucial path inducing cellular senescence downstream many tumor suppressor genes^18–22^.

In the present study, we explored the role of ASF1a in the immortal phenotype of cancer cells. We found that knockdown of ASF1a elicited DNA damage, thereby leading to growth arrest and senescence of HepG2 and LNCap cancer cells by activating the p53–p21^cip1^ axis. The TCGA data revealed a negative correlation between ASF1a and p21^cip1^ expression in HCC, prostate cancer (PCa), gastric cancer (GC), and breast cancer (BC). Moreover, higher ASF1a expression and lower p21^cip1^ expression predict a poor outcome in HCC patients, indicating the potential value of ASF1a in cancer as a prognostic biomarker and therapeutic target.

## Materials and methods

### Cell lines and cell culture

The wt p53-carrying HCC cell line HepG2, PCa line LNCaP, GC line AGS, BC line MCF-7, colon cancer line HCT116, HCT116-Cas9 and its variant HCT116 p53-/- subline, and normal human umbilical-derived mesenchymal stem cells (UMSCs) were used in this study. HepG2, LNCaP, and MCF-7 cells were cultured in RPMI-1640 medium (Thermo Fisher Scientific, Waltham, MA, USA) with 10% fetal bovine serum (Thermo Fisher Scientific), 100 U/ml penicillin, 100 μg/ml streptomycin, and 4 mM L-glutamine. AGS cells were cultured in F12 medium (Thermo Fisher Scientific). HCT116 cells and their variants were cultured in Dulbecco's modified Eagle's medium (Thermo Fisher Scientific). UMSCs were cultured in F12 medium with bFGF (10 ng/ml). In addition, two HCC cell lines, Huh.7 and Hep3B with mutant and null p53, respectively, were also included. All cells were incubated at 37 °C in a humidified 5% CO_2_ atmosphere.

### Clinical samples

Fifty-one patients with newly diagnosed HCC were recruited from Shandong University Second Hospital and Qilu Hospital, China. Fifty-one tumor specimens from these patients were collected immediately after surgery and stored in a −80 °C freezer or in TRIzol reagent (Life Technologies, Carlsbad, CA, USA) at −80 °C. Non-tumorous adjacent liver tissues were also available from 29 of these HCC patients. The HCC diagnosis was confirmed by histological examination. The study was approved by the Shandong University Second Hospital ethics committee.

### siRNA and transfections

ASF1a, p53, and negative control siRNAs were purchased from Thermo Fisher Scientific. We transfected cells with 20 nM siRNA in antibiotic-free Opti-MEM (Thermo Fisher Scientific) for 72 h using Lipofectamine 2000 (Thermo Fisher Scientific) according to the manufacturer's instruction. “Control” in the text refers to negative siRNA control. Sequences for these siRNAs are listed in Table S1.

### RNA extraction and quantitative real-time PCR

Total RNA was extracted from harvested cells or primary HCC tissues using TRIzol reagent (Life Technologies) according to the manufacturer’s instruction, and High Capacity cDNA Reverse Transcription Kit (Thermo Fisher Scientific) was used for the reverse transcription of total RNA into cDNA. SYBR Green PCR Master Mix (Life technologies) was used for quantitative real-time PCR (qPCR) reactions. The sequences of primers used in the qPCR assays are shown in Table S1. qPCR reactions were performed with QuantStudio 7 Flex Real-Time PCR system (Applied Biosystems, Walthan, MA, USA). Relative levels of target mRNAs were expressed as arbitrary units based on their CT values with normalization by β-2M expression.

### p21^cip1^ promoter reporters and luciferase activity assay

The pWWP-Luc (p21/WAF1 promoter, plasmid #16451) plasmid was obtained from Addgene (Middlesex, UK). The pGL3 Basic luciferase reporter vector was purchased from Promega (Madison, WI, USA). The Luciferase Assay System (Promega) was used to measure firefly and Renilla luminescence, and the Renilla signal was used for normalization. The luciferase activity in negative control cells was set as 1.00 (reference) for the comparison between negative control and ASF1a-depleted cell groups.

### Colony-formation assay

Seventy-two hours after ASF1a siRNA transfection, HepG2 and LNCaP cells were seeded in six-well plates (1000 cells/well). After 12 days of incubation (at 37 °C in a humidified 5% CO_2_ atmosphere), the colonies were fixed and stained with methanol and Giemsa buffer, respectively, and the colony numbers were counted. The number of colonies derived from the negative control cells was set as 100% (reference) for comparison between negative control and ASF1a-depleted cells.

### Cell cycle analysis

Cells were fixed with 70% ethanol at + 4 °C overnight and stained with a solution containing RNase A (0.5 μg) (Sigma-Aldrich; St. Louis, MO, USA) and propidium iodide (50 μg/ml) (Sigma-Aldrich). Cell cycle distribution was determined using flow cytometry. Kaluza software (Beckman Coulter, Indianapolis, IN, USA) was used for data analysis.

### β-Galactosidase staining

HepG2 and LNCaP cells were transfected with siRNAs and cultured for 8–10 days. Fresh medium was supplemented during this period. Cells were then rinsed with phosphate-buffered saline (PBS), fixed with 4% formaldehyde in PBS, and then incubated with freshly prepared senescence-associated β-Gal staining solution (Cellular Senescence Assay KAA002; Merck, Darmstadt, Germany) at 37 °C overnight. β-Galactosidase (β-gal)-positive cells were counted.

### Western blotting

RIPA lysis buffer (Cell signaling Technology (CST), Danvers, MA, USA) was used for protein extraction. Samples were run on sodium dodecyl sulphate-polyacrylamide gel electrophoresis and transferred to the polyvinylidene difluoride membrane (Bio-Rad, Hercules, CA, USA). The membrane was blocked with 5% non-fat milk for 2 h at room temperature and the primary antibodies were added. Membranes were incubated with the primary antibody at 4 °C overnight with gentle shaking. The secondary antibodies were added the next day, and Bio-rad ECL reagents (#1705062) were used to detect proteins on the blots. ChemiDoc™ MP Imaging System (Bio-Rad) was used for blot imaging. Quantification was done by using Image Lab™ Software (Bio-Rad). Actin was used for normalization. Target expression in negative control groups was set as 1 (reference) for comparison between control and ASF1a-depleted cell groups.

### Immunofluorescence staining

HepG2 and LNCaP cells were seeded on coverslips and transfected with ASF1a and control siRNAs, respectively. After treatment and culture, the cells were fixed in 4% formaldehyde and washed with PBS. After having blocked for 1 h in the blocking buffer (Dako, Santa Clara, CA, USA), the cells were incubated with the primary antibodies γH2AX and 53BP1 at 4 °C overnight. Triton-100 (Sigma-Aldrich) was used to increase the permeability of cellular and nuclear membranes. Alexa Fluor^TM^ 488 donkey anti-mouse IgG (H + L) and Alexa Fluor^TM^ 555 donkey anti-rabbit IgG (H + L) were used as the secondary antibodies, and 4-6-diamidino-2-phenylindole dihydrochloride (Cat no. H-1200; Vector Laboratories Inc., Burlingame, CA, USA) was used to detect the nuclei.

### Antibodies

ASF1a (C6E10) rabbit mAb (Cat no. 2990S) and p21^cip1^ (12D1) rabbit mAb (Cat no. 2947S) were purchased from CST. β-Actin (C4) HRP (sc-47778), p53 (DO-1) mouse mAb (sc-126), p-Histone H2A.X (Ser 139) mouse mAb (sc-517348), and HP1γ (F1) mouse mAb (sc-398562) were from Santa Cruz Biotechnology (Dallas, TX, USA). 53BP1 (A300-272A) was obtained from Bethyl Laboratories (Montgomery, TX, USA). Anti-trimethyl-histone H3 (Lys9) rabbit polyclonal Ab (Cat no. 07-442) was from Merck. TRF2 rabbit polyclonal Ab (NB110-57130) was from Novus Biologicals (Centennial, CO, USA). Goat anti-rabbit (Cat no. 170-6515) and goat anti-mouse (Cat no. 170-6516) IgG (H + L)-HRP conjugate secondary antibodies were from Bio-Rad. Donkey anti-mouse IgG (H + L) Alexa Fluor 488 (Cat no. A-21202) and Alexa Fluor^TM^ 555 donkey anti-rabbit IgG (H + L) secondary antibody (Cat no. A-31572) were bought from Thermo Fisher Scientific.

### Southern blot

HepG2 and LNcaP cells were transfected with negative control and ASF1a siRNAs and cultured for 72 h. Cells were harvested and genomic DNA was extracted with QIAamp DNA Blood Mini Kit (Cat no 51104; Qiagen, Hilden, Germany). Southern blot was performed by using Telo *TAGGG* Telomere Length Assay (Merck; Cat no. 12 209 136 001) according to the manufacturer’s instructions.

### Statistical analysis

Experimental quantitative data were obtained in biological replicates and shown as means ± standard deviation if not otherwise indicated. Student’s *t* test was used for analyses of differences between experiment groups. The Cancer Genome Atlas (TCGA) data of ASF1a and p21^cip1^ expression were downloaded via cBioPortal (http://www.cbioportal.org) (January, 2018). Pearson correlation coefficient was used to assess the relationship between the two variables. ASF1a and p21^cip1^ survival analysis was done by GEPIA (http://gepia.cancer-pku.cn), which retrieved TCGA data. Disease-free and overall survival (DFS and OS) was illustrated by Kaplan–Meier plots, and the log-rank test was used to compare survival distributions (between groups). All the tests were two-tailed and computed using GraphPad Prism 6.0c (GraphPad Software Inc.). *P* values< 0.05 were considered as statistically significant.

## Results

### ASF1a is overexpressed in a variety of primary human tumors

**Fig. 1:**
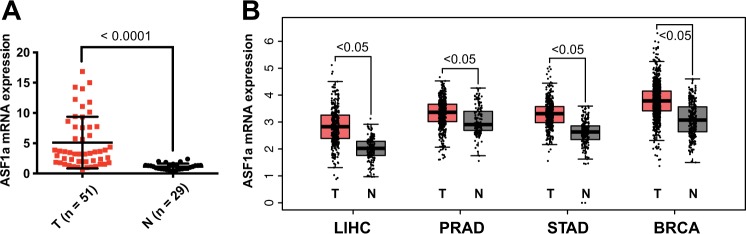
ASF1a is overexpressed in a variety of primary human tumors. **a** Relative ASF1a mRNA levels were determined using qPCR in hepatocellular carcinoma (HCC) (T: tumor, *n* = 51) samples and adjacent non-tumorous liver tissues (*N*: non-tumorous, *n* = 29) (data are presented as the mean ± SD; *P* < 0.0001). **b** ASF1a mRNA expression (transcripts per million in log scale) in liver hepatocellular carcinoma (LIHC) (T, *n* = 369; N, *n* = 160), prostate adenocarcinoma (PRAD) (T, *n* = 408; N, *n* = 152), stomach adenocarcinoma (STAD) (T, *n* = 408; N, *n* = 221), and breast invasive carcinoma (BRCA) (T, *n* = 1085; N, *n* = 291) (data are presented as the mean ± SD; *P* < 0.05). qPCR, quantitative real-time PCR

We first determined ASF1a mRNA expression in 51 primary HCC tumors and 29 adjacent non-tumorous liver tissues. ASF1a mRNA was overexpressed in most tumors (5.11 ± 4.26 vs 1.14 ± 0.50 for tumors vs non-tumorous tissues, respectively; *P* < 0.0001) (Fig. 1a). ASF1a protein expression in collected tumor samples was also higher than that in the non-tumorous samples (Supplementary Fig. S1A). We then analyzed the data from the TCGA (https://cancergenome.nih.gov) and GTEx (https://gtexportal.org/home/) databases, and the results were documented in Fig. 1b: ASF1a was overexpressed in HCC (liver hepatocellular carcinoma, LIHC), PCa, GC, and BC tumor tissues compared with their non-tumorous counterparts. Data from Oncomine (https://www.oncomine.org/resource/login.html) also exhibited stronger ASF1a expression in HCC, PCa, GC, and BC, which was in accordance with the results obtained from TCGA and GTEx (Fig. S1B).

### ASF1a knockdown leads to growth arrest and cellular senescence in wt p53-carrying HCC and PCa cells

**Fig. 2:**
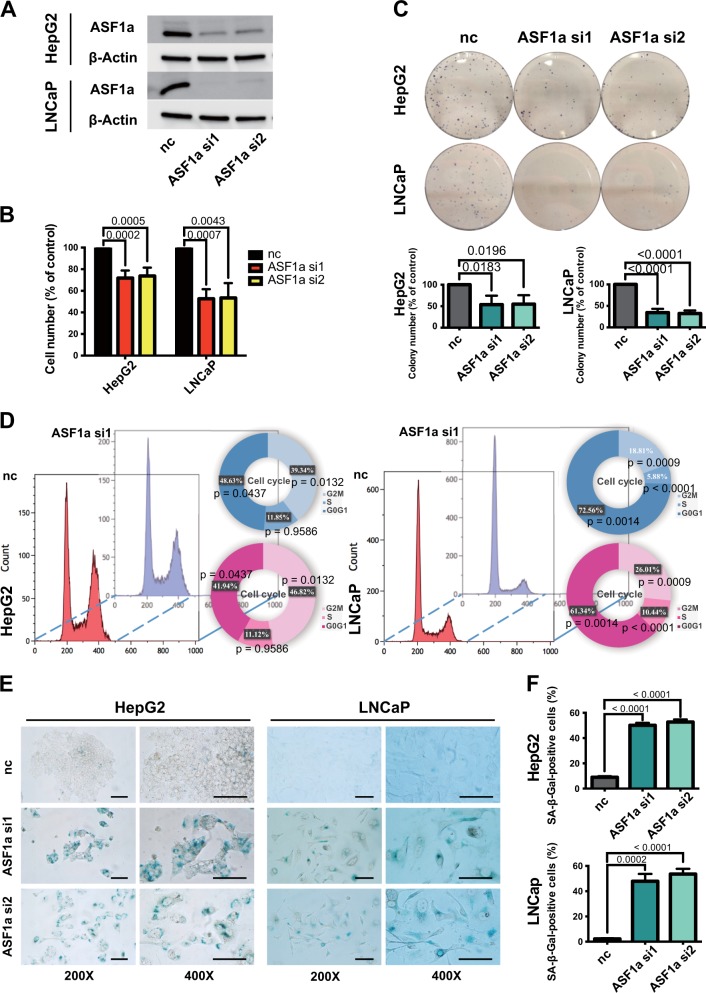
ASF1a knockdown leads to growth arrest and cellular senescence in wild-type p53-carrying HCC and PCa cells. **a** Western blot was performed for the validation of ASF1a siRNA efficiency in HepG2 and LNCaP cells. ASF1a si1 and ASF1a si2, two different ASF1A siRNAs, were used in this study. **b** The cell number of control (nc siRNA) and ASF1a knockdown (ASF1a si1/si2) groups in HepG2 and LNCaP cells 72 h after siRNA transfection. Cells in negative control groups were set as 100% (reference). The siASF1a-transfected groups showed a significant decrease in cell counts compared with control cells (data are presented as the mean ± SD of four independent experiments in HepG2 and three independent experiments in LNCaP, respectively; *P* values are shown in each panel). **c** Colony-formation assay of HepG2 and LNCaP cells. The clonogenic abilities of HepG2 and LNCaP cells were decreased upon ASF1a knockdown. Quantification is shown at the bottom (data are presented as the mean ± SD of three independent experiments in HepG2 and four independent experiments in LNCaP, respectively; *P* values are shown in each panel). Colonies in control groups were set as 100% (reference). **d** Cell cycles of HepG2 and LNCaP recorded by propidium iodide (PI) staining. Cell cycles in control groups are shown with red peaks and cell cycles in ASF1a knockdown groups are shown with blue peaks. Proportions of G0/G1, S, and G2/M phases are presented in pie charts (data presented are of three independent experiments in HepG2 and four independent experiments in LNCaP; *P* values are shown in each panel). **e** β-Gal staining of HepG2 and LNCaP cells. Senescent cells were stained with blue color (scale bar: 100 μm). **f** Quantification of β-gal staining-positive HepG2 and LNCaP cells (data are presented as the mean ± SD value of three independent experiments for HepG2 and LNCaP, respectively). HCC, hepatocellular carcinoma; PCa, prostate cancer

We chose the HCC cell line HepG2 and the PCa cell line LNCaP to explore ASF1a’s function. First, we introduced ASF1a siRNA into HepG2 and LNCaP cells to inhibit ASF1a expression. ASF1a siRNA knockdown efficiency was verified by western blot (Fig. 2a). We then assessed the proliferation rate of these ASF1a-depleted cells (Fig. 2b). The cell number was significantly lower in siASF1a-treated cells than in the control ones (100 ± 0.0, 71.8 ± 7.0, and 73.7 ± 7.8% for control, ASF1a si1 and ASF1a si2 in HepG2 cells, respectively and 100 ± 0.0, 52.6 ± 8.8, and 53.3 ± 13.8% for control, ASF1a si1 and ASF1a si2 in LNCaP cells, respectively). The observed cell number decrease was not due to significant cell death, because the viability was >97 and >86% for all the HepG2 and LNCaP groups, respectively (Supplementary Fig. S2A). We further performed colony-formation assays (Fig. 2c). Both HepG2 and LNCaP cells treated with siASF1a formed much fewer colonies than did control cells (100 ± 0.0, 53.7 ± 20.8, and 54.8 ± 20.8% for control, ASF1a si1 and ASF1a si2 in HepG2 cells, respectively and 100 ± 0.0, 34.4 ± 8.8, and 32.3 ± 7.0% for control, ASF1a si1 and ASF1a si2 in LNCaP cells, respectively), indicating that ASF1a inhibition greatly impaired the clonogenic potential. Seventy-two hours after ASF1a siRNA transfection, HepG2 and LNCaP cells were harvested and cell cycle was analyzed using flow cytometry (Fig. 2d). Both HepG2 and LNCaP cells with ASF1a silencing accumulated at G0/G1 phase (siASF1a vs control, 50.7 ± 3.7 vs 43.3 ± 2.4%, *P* = 0.04 and 72.6 ± 3.8 vs 61.3 ± 1.2%, *P* = 0.001, for HepG2 and LNCaP, respectively) and decreased at G2/M phase (siASF1a vs control, 38.3 ± 1.9 vs 45.7 ± 2.3%, *P* = 0.001 and 18.8 ± 2.1 vs 26.0 ± 1.0%, *P* = 0.0009, for HepG2 and LNCaP, respectively). Cells became larger and flatter following ASF1a siRNA transfection in both HepG2 and LNCaP, which indicated a senescence-like morphology. We, thus, performed β-gal staining for senescence evaluation (Fig. 2e, f). As shown in Fig. 2e, blue β-gal staining was readily seen in siASF1a-treated cells, while hardly detected in control cells, implying that knockdown of ASF1a led to senescence of HepG2 and LNCaP cells. Quantification is shown in Fig. 2f (negative control vs ASF1a si1 vs ASF1a si2, 9.0 ± 0.8 vs 50.2 ± 1.9% vs 52.7 ± 2.0 and 2.2 ± 0.5% vs 47.9 ± 5.8 vs 53.6 ± 4.2%, for HepG2 and LNCaP cells, respectively; *P* values are shown in the appropriate panels).

### Cellular senescence is induced via the p53–p21^cip1^ pathway in ASF1a-depleted cells

**Fig. 3:**
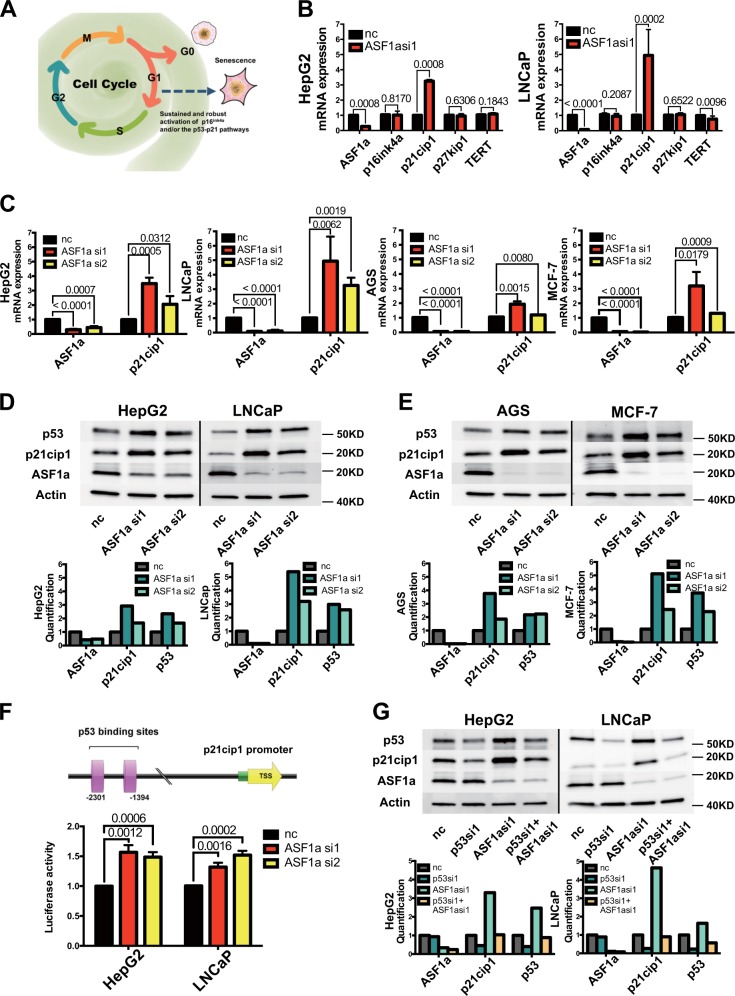
Cellular senescence is induced via the p53/p21^cip1^ pathway in ASF1a-depleted cells. **a** The schematic expression showing that RB/p16^ink4^ and p53/p21^cip1^ cascades are the two major pathways involved in cellular senescence. **b** A screen of four cellular senescence-associated genes in HepG2 and LNCaP cells. p16^ink4^, p21^cip1^, p27^kip1^, and TERT mRNA expression in control (nc) and ASF1a knockdown (ASF1a si1) groups is shown in the bar charts (data are presented as the mean ± SD; *P* values are shown in each panel). **c** Loss of ASF1a increased p21^cip1^ mRNA expression in HepG2, LNCaP, AGS, and MCF-7 cells (data are presented as the mean ± SD, at least three independent experiments for each cell line, respectively; *P* values are shown in each panel). **d** Loss of ASF1a increased p21^cip1^ and p53 protein expression in HepG2 and LNCaP cells. Quantification is shown at the bottom. **e** Loss of ASF1a increased p21^cip1^ and p53 protein expression in AGS and MCF-7 cells. Quantification is shown at the bottom. **f** The schematic of p21^cip1^ (CDKN1A) promoter and luciferase activity. An increased luciferase activity was observed upon ASF1a knockdown in both HepG2 and LNCaP cells (data are presented as the mean ± SD of three independent experiments for HepG2 and LNCaP, respectively; *P* values are shown in each panel). The landscape figure was drawn using the GPS 3.0 tool (http://gps.biocuckoo.org). **g** Knockdown of p53 compromises ASF1a depletion-induced p21^cip1^ accumulation, as assessed using immunoblotting. Quantification is shown at the bottom

**Fig. 4:**
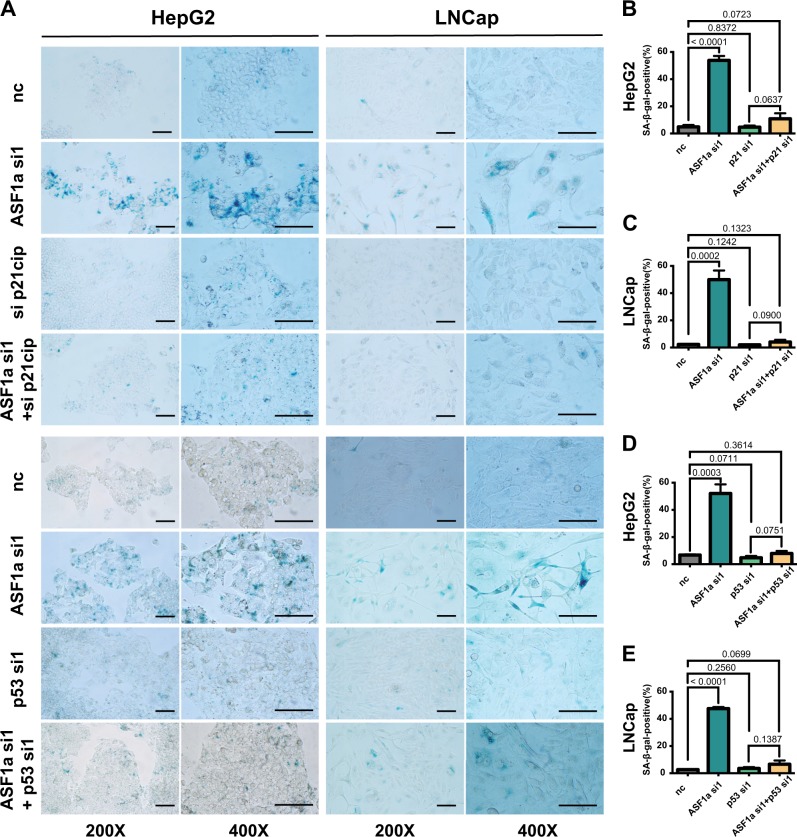
Cellular senescence occurring in ASF1a-inhibited cells is rescued by p21^cip1^ or p53 loss. **a** β-Gal staining in HepG2 and LNCaP cells. Senescent cells are stained with blue color (scale bar: 100 μm). **b**–**e** Quantifications of figure (**a**) (data are presented as the mean ± SD of three independent experiments for HepG2 and LNCaP, respectively; *P* values are shown in each panel)

Since senescent cells quit from cell cycle as a result of the activation of sustained and robust p16^ink4^–pRB and/or p53–p21^cip1^ pathways, we did a screen of four cellular senescence-associated genes to investigate the underlying mechanisms (Fig. 3a, b). The qPCR results showed that p21^cip1^ mRNA level was significantly elevated upon ASF1a knockdown (siASF1a vs control, 3.25 ± 0.09 vs 1.00 ± 0.01, *P* = 0.0008 and 4.94 ± 1.70 vs 1.02 ± 0.04, *P* = 0.0002, for HepG2 and LNCaP cells, respectively), while there were no detectable changes in p16^ink4a^, p27^kip1^, and TERT expression. Cellular senescence markers PML and SERPINI1 were also up-regulated following ASF1a knockdown in LNCaP cells, which was an indicator of p53 activation (Supplementary Fig. S2B). The histone variant macroH2A was positive in ASF1a knockdown LNCap cells (Supplementary Fig. S2C). We further verified the effect of ASF1a depletion on p21^cip1^ expression at both mRNA and protein levels in wild-type (wt) p53-carrying cell lines HepG2 and LNCaP, as well as in AGS and MCF-7 cells (Fig. 3c–e), which was coupled with increased p53 expression (Fig. 3d, e). As p53 activates p21^cip1^ transcriptionally by binding to its consensus motif (-2301 and -1394 from the transcription start site) on the p21^cip1^ distal promoter, we wanted to determine whether this is the case in ASF1a-depleted cells. The pWWP luciferase reporter vector, containing the 2.4 kb fragment upstream the transcription start site of the *CDKN1A* gene, was transfected into HepG2 and LNCaP cells 24 h post-ASF1a knockdown and these cells were then incubated for another 48 h. The results showed an increased p21^cip1^ promoter activity in siASF1a-treated cells (control vs ASF1a si1 and ASF1a si2: 1.00 ± 0.00 vs 1.57 ± 0.12 and 1.49 ± 0.08, and 1.00 ± 0.01 vs 1.32 ± 0.07 and 1.52 ± 0.07, for HepG2 and LNCaP cells, respectively) (Fig. 3f). To further evaluate p53 effect on p21^cip1^ accumulation caused by ASF1a depletion, we knocked down p53 and ASF1a simultaneously. As shown in Fig. 3g, p53 depletion attenuated ASF1a knockdown effect on p21^cip1^ augmentation, which indicates the causal role of p53 in ASF1a-mediated p21^cip1^ regulation. We then performed β-gal staining in HepG2 and LNCaP cells transfected with ASF1a and p21^cip1^ siRNAs (negative control vs ASF1a si1 vs sip21^cip1^ vs ASF1a + sip21^cip1^) for 10 days. We found that the senescence phenotype observed in ASF1a-inhibited cells was rescued by p21^cip1^ knockdown. p53 loss could also attenuate the ASF1a knockdown-induced cellular senescence phenotype (Fig. 4a). Quantification is shown in Fig. 4b–e. These data unveil a potential ASF1a-p53-p21^cip1^ regulatory axis in ASF1a inhibition-induced cellular senescence in HepG2 and LNCaP cells.

**Fig. 5:**
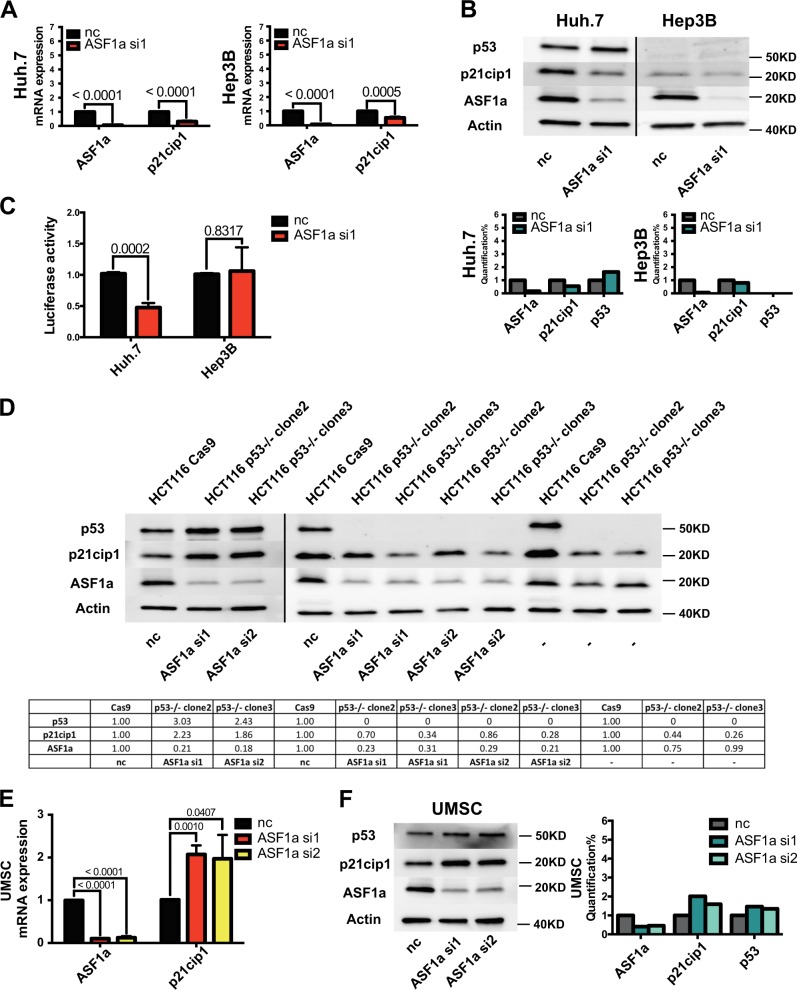
ASF1a inhibition fails to induce p21^cip^ expression in p53 mutant or null HCC cells. **a** Knockdown of ASF1a decreased p21 mRNA expression in the p53 mutant cell line Huh.7 and p53-null Hep3B cells (data are presented as the mean ± SD of three independent experiments for Huh.7 and Hep3B, respectively; *P* values are shown in each panel). **b** Western blot showed unchanged or even diminished p21^cip1^ expression after ASF1a knockdown in Huh.7 and Hep3B cells. Quantification is shown at the bottom. **c** Luciferase assay showed a reduced p21^cip1^ promoter activity in ASF1a depletion groups in Huh.7 cells while no alteration in Hep3B cells (data are presented as the mean ± SD of three independent experiments for Huh.7 and Hep3B, respectively; *P* values are shown in each panel). **d** Western blot showed accumulated and diminished p21^cip1^ expression after ASF1a knockdown in control Cas9 HCT116 and CRISPR-Cas9-edited p53-/- HCT116 cells, respectively. Quantification is shown at the bottom. **e** Loss of ASF1a increased p21^cip1^ mRNA expression in normal human umbilical-derived mesenchymal stem cells (UMSCs) (data are presented as the mean ± SD of three independent experiments; *P* values are shown in the panel). **f** Western blot showed slightly increased p53 and p21^cip1^ protein levels in ASF1a knockdown UMSCs. Quantification is shown on the right panel. HCC, hepatocellular carcinoma

To further explore whether ASF1a inhibition-induced p21^cip1^ accumulation was p53-dependent, we knocked down ASF1a in two HCC cell lines Huh.7 (p53 mutant) and Hep3B (p53 null), and then assessed p21^cip1^ mRNA and protein expression. Unexpectedly, qPCR and western blot results showed a significant decline in p21^cip1^ expression (Fig. 5a, b; control vs ASF1a si1, 1.00 ± 0.01 vs 0.31 ± 0.06 and 1.00 ± 0.01 vs 0.53 ± 0.08, for Huh.7 and Hep3B cells, respectively). Consistent with p21^cip1^ expression, ASF1a depletion led to a diminished p21^cip1^ promoter activity in Huh7 cells, while there were no significant alterations in Hep3B cells (Fig. 5c; control vs ASF1a si1, 1.02 ± 0.02 vs 0.48 ± 0.07 and 1.01 ± 0.02 vs 1.06 ± 0.38, for Huh.7 and Hep3B cells, respectively).

To further evaluate the role of p53 in ASF1a inhibition-associated p21^cip1^ activation, we introduced CRISPR-Cas9-edited HCT116 cells, which originally express wt p53. Both p21^cip1^ and p53 were up-regulated in HCT116-Cas9 cells after ASF1a knockdown (Fig. 5d, left). p53 was knocked out in HCT116 p53-/- cells by using CRISPR-Cas9 system. p21^cip1^ accumulation could not be observed after ASF1a knockdown in these p53-/- cells (Fig. 5d, right). Additionally, in normal human UMSCs, already abbreviated p53 and p21^cip1^ were up-regulated to a certain extent upon ASF1a knockdown, although not as robust as seen in wt p53-carrying cancer cell lines following ASF1a depletion (Fig. 5e, f).

### Silencing ASF1a triggers DNA damage response

**Fig. 6:**
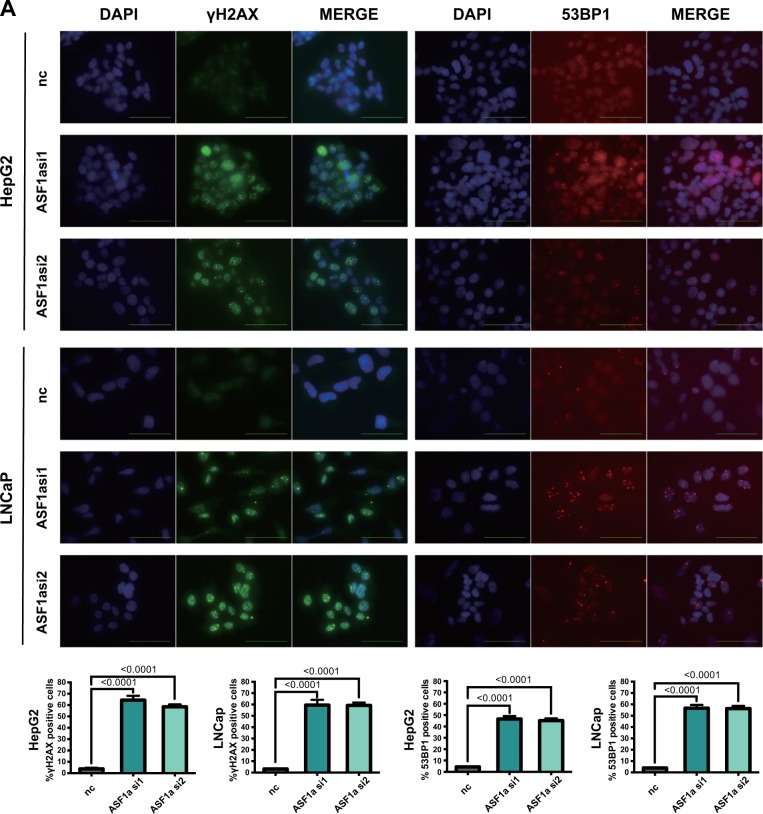
Silencing ASF1a triggers DNA damage response. **a** Immunofluorescence staining of control (nc) and ASF1a knockdown (ASF1a si1/si2) groups of HepG2 and LNCaP cells. Nuclei were stained with DAPI (blue signals). γH2AX and 53BP1 were stained with specific antibodies (green and red signals, respectively; scale bar: 50 μm). Quantification is shown at the bottom (data are presented as the mean ± SD value of three independent experiments for HepG2 and LNCaP, respectively). Senescence-associated heterochromatin foci (SAHF) were not detected by using DAPI staining. DAPI, 4-6-diamidino-2-phenylindole dihydrochloride

Since p53 is induced by DDR, we sought to determine whether ASF1a knockdown causes DNA damage, thereby triggering DDR and p53 accumulation. We performed immunofluorescence staining to examine the formation of γH2AX and 53BP1 foci, two typical biomarkers for DNA damage (Fig. 6a, γH2AX: green signals; 53BP1: red signals). γH2AX and 53BP1 foci robustly increased in ASF1a-depleted cell lines (γH2AX: control vs ASF1a si1 and ASF1a si2, 3.8 ± 1.0 vs 64.4 ± 3.8% and 58.5 ± 2.1 and 3.2 ± 0.5% vs 59.5 ± 4.6 and 59.2 ± 1.5%, for HepG2 and LNCaP, respectively; 53BP1: control vs ASF1a si1 and ASF1a si2, 4.3 ± 0.2 vs 46.6 ± 2.6% and 45.2 ± 2.0 and 4.0 ± 0.2% vs 56.6 ± 2.9 and 56.3 ± 2.3%, for HepG2 and LNCaP, respectively). γH2AX and 53BP1 expression was elevated after knocking down p53 and both p53 and ASF1a (Supplementary Fig. S3A). We could not detect the senescence-associated heterochromatin foci (SAHF) by using the 4-6-diamidino-2-phenylindole dihydrochloride staining in HepG2 and LNCaP (Fig. 6a); however, H3K9me3 and HP1γ proteins were up-regulated after ASF1a knockdown (Supplementary Fig. S3B). Since telomeres are the targets of DDR associated with cellular senescence^23^, we thus determined whether ASF1a inhibition results in telomeric damage. We performed immunofluorescence for telomere-associated protein TRF2 and DDR marker γH2AX in HepG2 and LNCaP cells transfected with negative control and ASF1a siRNAs. The confocal captured images showed the colocalization of the two markers (Supplementary Fig. S4A; white arrows). We also checked if telomere shortening happened in ASF1a inhibition-induced cellular senescence by performing Southern blot. The result showed no significant change in telomere length in ASF1a knockdown groups in both HepG2 and LNCaP cells (Supplementary Fig. S5A).

### ASF1a and p21^cip1^ expression is negatively correlated with each other and predicts patient outcomes in HCC

**Fig. 7:**
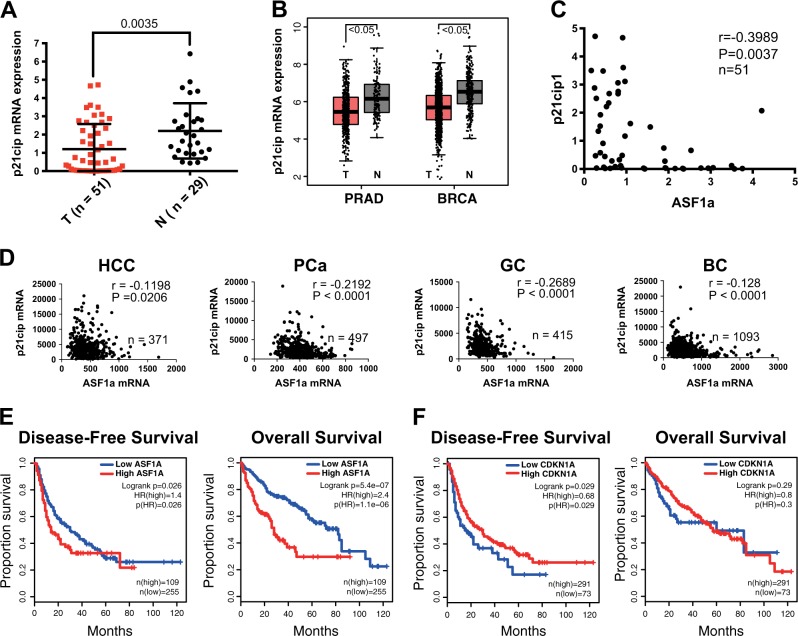
ASF1a and p21^cip^ expression is negatively correlated with each other and serves as prognostic factors in HCC. **a** Relative p21^cip1^ mRNA levels were assessed by qPCR in HCC (T: tumor, *n* = 51) patient samples and non-tumorous adjacent liver tissue (N: non-tumorous, *n* = 29) (data are presented as the mean ± SD, *P* = 0.0035). **b** Data from TCGA and GTEx databases revealed a lower p21^cip1^ mRNA expression (transcripts per million in log scale) in tumor tissues compared with normal tissues in PRAD and BRCA (data are presented as the mean ± SD, *P* < 0.05). **c** A negative correlation between ASF1a and p21^cip1^ mRNA expression in HCC patient samples (*r* = -0.3989, *P* = 0.0037, *n* = 51). **d** A negative correlation between ASF1a and p21^cip1^ mRNA expression in HCC, PCa, GC, and BC, respectively. Data were from the TCGA database. **e** Higher ASF1a expression is a predictor of shorter disease-free survival (DFS) and overall survival (OS) in HCC patients. A cutoff of 0.7 was used to categorize patients into low and high ASF1a expression groups (Mantal–Cox test was used in the survival analysis). Hazard ratio (HR) was presented in each panel, respectively. **f** Higher p21^cip1^ expression is a predictor for longer disease-free survival. A cutoff of 0.2 was used to split patients into low and high p21^cip1^-expression groups (Mantal–Cox test was used in the survival analysis). HR was presented in each panel, respectively. BC, breast cancer; GC, gastric cancer; HCC, hepatocellular carcinomaq; PCa, prostate cancer; qPCR, quantitative real-time PCR; TCGA, The Cancer Genome Atlas

We finally wanted to determine the relationship between ASF1a and p21^cip1^ expression and its potential clinical significance. First, we examined p21^cip1^ mRNA expression in our HCC patient samples, and p21^cip1^ expression was down-regulated in tumor tissues compared with their normal counterparts (Fig. 7a; 1.20 ± 1.38 vs 2.20 ± 1.51 for tumors vs non-tumorous tissues, *P* = 0.0035). TCGA and GTEx data showed significantly decreased p21^cip1^ transcripts in tumor tissues in prostate cancer (PRAD) and breast cancer (BRCA) (Fig. 7b). We analyzed the Oncomine data and found down-regulated p21^cip1^ expression in HCC, PCa, GC, and BC tumors (Supplementary Fig. S1C). A negative correlation between ASF1a and p21^cip1^ expression could be drawn from our HCC patient samples available (Fig. 7c). A negative correlation between ASF1a and p21^cip1^ expression was also observed in HCC (*r* = -0.1198, *P* = 0.0206, *n* = 371), PCa (*r* = -0.2192, *P* < 0.0001, *n* = 497), GC (*r* = -0.2689, *P* < 0.0001, *n* = 415), and BC (*r* = -0.1280, *P* < 0.0001, *n* = 1093) derived from the TCGA dataset (Fig. 7d). The prognostic value of both ASF1a and p21^cip1^ in HCC patients was determined by analyzing the TCGA dataset (Fig. 7e, f). Based on a cutoff value of 0.7, 109 patients had higher ASF1a-expressing tumors, while those with values lower than 0.7 were considered the low-expression cohort. The Mantel–Cox test (log-rank) revealed that higher ASF1a expression was significantly associated with both shorter DFS and OS (Fig. 7e). A cutoff value of 0.2 was used in p21^cip1^-associated DFS and OS analysis. The Mantel–Cox test (log-rank) shows that higher p21^cip1^ expression predicted a longer DFS (Fig. 7f). Because the biological functions of ASF1a and p21 do not totally overlap with each other, they unlikely match perfectly in predicting patient survival. Nevertheless, both predict DFS in HCC, which, from a clinical or therapeutic point of view, should be a more relevant prognostic variable than OS. The hazard ratio is also presented in each panel, showing the association of ASF1a and p21^cip1^ with survival in HCC.

## Discussion

ASF1a is originally recognized as a histone chaperone that interacts with newly synthesized histone H3–H4 dimers and passes the dimers to the histone chaperone CAF-1 to assemble nucleosomes after DNA replication or repair^24–27^. ASF1a also cooperates with acetyltransferase CBP/p300 to acetylate H3 at lys56 position, which is regarded as a marker for chromatin disassembly and active transcription^28^. Recently, ASF1a was reported to be required for the maintenance of cell pluripotency and reprogramming^4^. These findings indicate that ASF1a has transcriptional activation and stemness-promoting functions. We have previously shown ASF1a as an oncogenic factor in gastrointestinal cancer. ASF1a interacts with the transcription factor β-catenin and activates the downstream genes cyclin D1, myc, Lgr5, and ZEB1, promoting cancer development and progression^29^. In the present study, we observed that ASF1a was required for the sustained proliferation of cancer cells, whereas its inhibition led to cellular senescence by triggering DNA damage and thereby up-regulating the p53/p21^cip1^ pathway. By analyzing the TCGA and GTEx data, we further show that ASF1a is overexpressed in 20 different types of cancers compared with their non-tumorous counterparts^30^ (Supplementary Fig. S6A). Moreover, higher ASF1a expression is significantly associated with a poor patient outcome in HCC patients. These findings collectively indicate that the aberrant up-regulation of ASF1a is widespread in human malignancies and contributes to the unlimited proliferation of cancer cells.

There have been a number of investigations regarding ASF1a’s role in cellular senescence. During cellular senescence, the region containing proliferation-promoting genes are condensed into the transcriptionally silent heterochromatin named SAHF^31–34^, the formation of which requires ASF1a and HIRA (histone cell cycle regulation defective homolog A)^31,35^. The involvement of ASF1a in SAHF formation seems to make the ASF1a regulatory network more complicated in senescent cells. In our study, we did not observe SAHF formation in ASF1a-depleted cells. There are several lines of evidence showing that SAHF formation and senescence are not always coupled. First, SAHF formation is cell content-dependent^36^. For example, SAHF is observed in the senescent human embryonic fibroblast cell lines IMR90 and WI38 but not in the primary human foreskin fibroblast cell line BJ^32^. Second, Raffaella and colleagues report that SAHF is preferentially formed in oncogene-triggered cellular senescence relying on the p16^ink4^ pathway but not in replicative cellular senescence induced by DDR/p53/p21^cip1^ signaling^37^. In addition, it has been well established that telomerase activation and induction of TERT expression are required for cancer cells to overcome the senescence barrier and to achieve an immortal phenotype^38^, while ASF1a inhibition was previously shown to contribute to diminished TERT expression in cancer cells. In the present study, however, we observed no detectable changes in TERT expression in ASF1a-depleted HepG2 and LNCaP cells. Moreover, telomere length did not shorten in these cells. It is thus evident that cellular senescence resulting from ASF1a knockdown is unrelated to telomerase/TERT expression or telomere length.

ASF1a plays a role in DNA repair: ASF1a-mediated H3K56 acetylation (H3K56ac) is required for nucleosome reassembly after DNA damage to facilitate DNA repair^39^. ASF1 promotes checkpoint recovery after DNA damage repair^40^. ASF1a and ATM can also modulate UV-induced cell-cycle checkpoint recovery^41^. Notably, ASF1a directly participates in DNA double-strand break repair, which highlights a novel role of ASF1a independent of a classic histone chaperone function^26^. Lee and colleagues report that ASF1a is required for 53BP1 recruitment and that there are less 53BP1 foci in ASF1a-depleted U2OS cells compared with the control ones upon DNA damage^26^. However, we observed more 53BP1 foci after ASF1a knockdown. Likewise, the ASF1a-related DNA repair mechanism might differ between different cell lines with different ASF1a expression or genetic backgrounds.

Our results suggest that DDR and p21^cip1^ induction mediated by ASF1a inhibition is p53-dependent. Intriguingly, the mutant p53-harboring Huh7 cells exhibited diminished p21^cip1^ expression while p53-null HepG3 cells had no detectable changes in p21^cip1^ expression upon ASF1a depletion. It is currently unclear why ASF1a inhibition leads to opposite consequences in cells with different p53 backgrounds. Likely, the gain of function of mutant p53 plays a dominant role in controlling p21^cip1^ expression. These findings may be both biologically and clinically important.

**Fig. 8:**
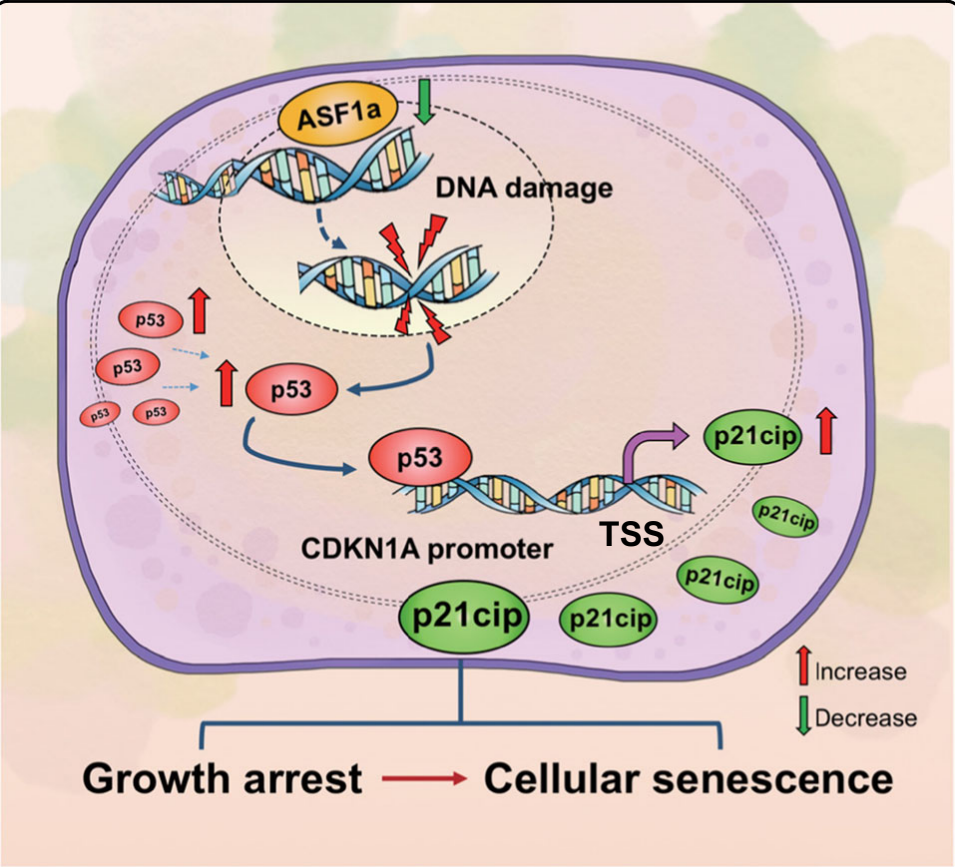
The schematic of the tentative mechanism underlying cancer cell senescence mediated by ASF1a inhibition

In summary, our findings indicate ASF1a as a crucial oncogenic factor aberrantly overexpressed in a variety of cancers. Knockdown of ASF1a induces growth arrest and cellular senescence by activating a DDR/p53/p21^cip1^ pathway in wt p53 HCC and PCa cell lines (Fig. 8). Both ASF1a and p21^cip1^ may serve as prognostic factors in HCC. The results support the concept that ASF1a is a potential novel target in cancer treatment.

## Supplementary information

Supplementary Information

## Data Availability

The analyzed datasets presented in the current study are available in TCGA (https://cancergenome.nih.gov), GTEx (https://gtexportal.org/home/), and Oncomine (https://www.oncomine.org/resource/login.html) repositories.
