# Supplementary material for: ASF1a inhibition induces p53-dependent growth arrest and senescence of cancer cells
Source: Cell Death Dis. 2019 Jan 28;10(2):76. doi: 10.1038/s41419-019-1357-z (PMC6349940; doi:10.1038/s41419-019-1357-z)
Supplement: Supplementary file 1 — Supplementary Information [file 41419_2019_1357_MOESM1_ESM.pdf]

Supplementary Information:

Figure S1: ASF1a protein expression in HCC samples and ASF1a and p21^{cip1} mRNA expression in primary tumor tissues derived from Oncomine database. (A) ASF1a protein expression in HCC samples (Quantification is on the right). (B)ASF1a was overexpressed in HCC, PCa, GC and BC tissues compared with non-tumorous tissues. (C) Lower p21^{cip1} was expressed in HCC, PCa, GC and BC tissues compared with their non-tumorous counterparts.

Figure S2: (A) Viability (number of live cells/number of total cells) of control (con) and ASF1a knockdown (ASF1a si1/si2) groups in HepG2 and LNCap cells. Cell numbers in control groups were set as 100% (reference) for comparison between control and ASF1a-depleted cells. (B) PML and SERPINI mRNA expression in LNCap cells (data are presented as the mean ± SD value of three independent experiments; p values are shown in the panel). (C) Histone variant macroH2A mRNA expression in LNCap cells (data are presented as the mean ± SD value of three independent experiments; p value is shown in the panel).

Figure S3: (A) Western blot result of γH2AX and 53BP1 expression in negative control, p53 knockdown and p53 knockdown + ASF1a knockdown HepG2 and LNCap cells. Quantification is at the bottom. (B) Western blot result of H3K9me3 and HP1γ expression in negative control and ASF1a knockdown HepG2 and LNCap cells. Quantification is at the bottom.

Figure S4: Confocal images of TRF2 and γH2AX colocalization in HepG2 and LNCap cells. Red signals: TRF2; Green signals: γH2AX. White arrows show the colocalization (scale bar: 10μm).

Figure S5: (A) Southern blot result shows telomere length in HepG2 and LNCap cells transfected with negative control and ASF1a siRNAs.

Figure S6: (A) ASF1a was overexpressed in 20 cancer types according to the TCGA data analyses.

Table S1. Primers and siRNA sequences used in the present study

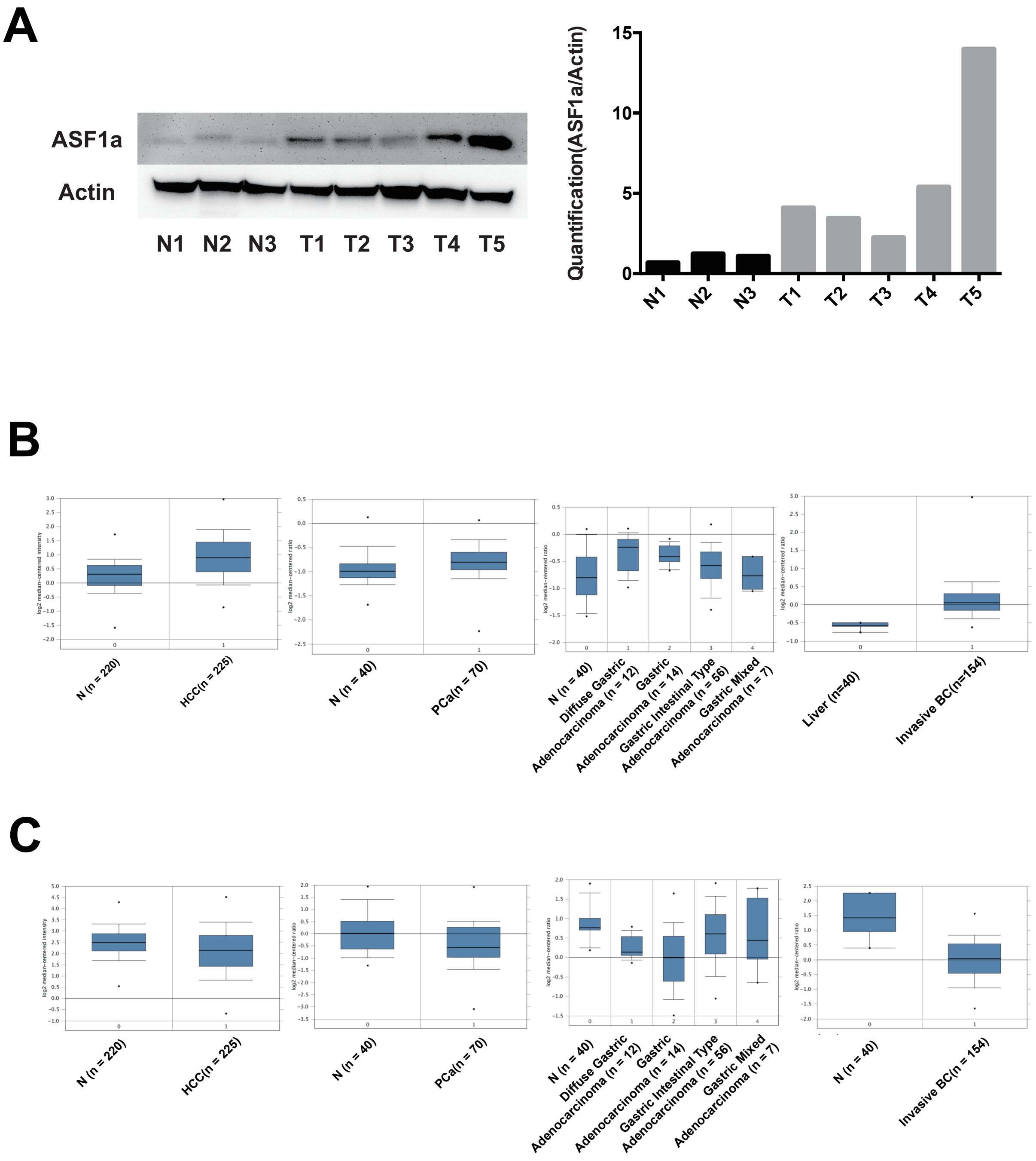
A
ASF1a
Actin
N1 N2 N3 T1 T2 T3 T4 T5
Quantification(ASF1a/Actin)
15
10
5
0
N1 N2 N3 T1 T2 T3 T4 T5
B
N (n = 220)
HCC(n = 225)
N (n = 40)
PCa(n = 70)
N (n = 40)
Diffuse Gastric Adenocarcinoma (n = 12)
Gastric Adenocarcinoma (n = 14)
Gastric Intestinal Type Adenocarcinoma (n = 56)
Gastric Mixed Adenocarcinoma (n = 7)
Liver (n=40)
Invasive BC(n=154)
C
N (n = 220)
HCC(n = 225)
N (n = 40)
PCa(n = 70)
N (n = 40)
Diffuse Gastric Adenocarcinoma (n = 12)
Gastric Adenocarcinoma (n = 14)
Gastric Intestinal Type Adenocarcinoma (n = 56)
Gastric Mixed Adenocarcinoma (n = 7)
N (n = 40)
Invasive BC(n = 154)

Fig. S1

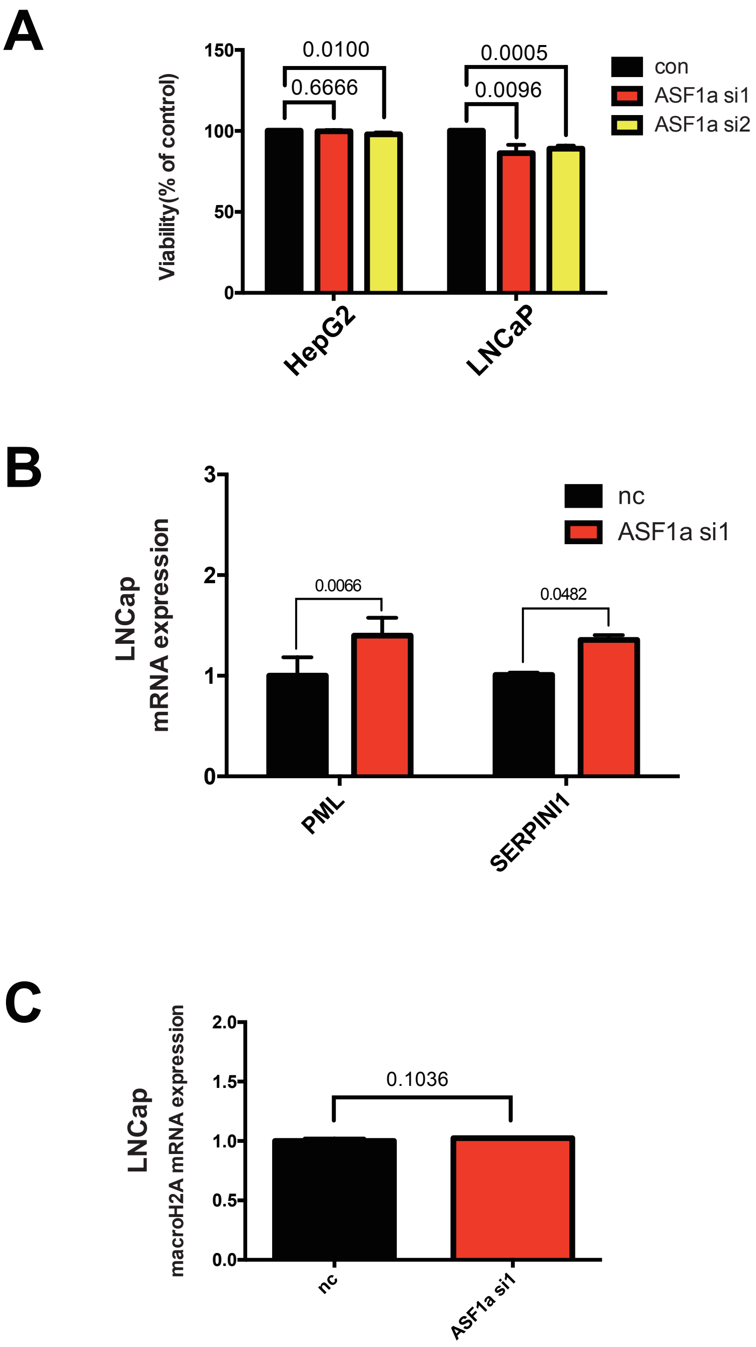
A
Viability(% of control)
150
100
50
0
0.0100
0.6666
0.0005
0.0096
con
ASF1a si1
ASF1a si2
HepG2
LNCaP
B
LNCap
mRNA expression
3
2
1
0
nc
ASF1a si1
0.0066
0.0482
PML
SERPINI1
C
LNCap
macroH2A mRNA expression
2.0
1.5
1.0
0.5
0.0
0.1036
nc
ASF1a si1

Fig. S2

A

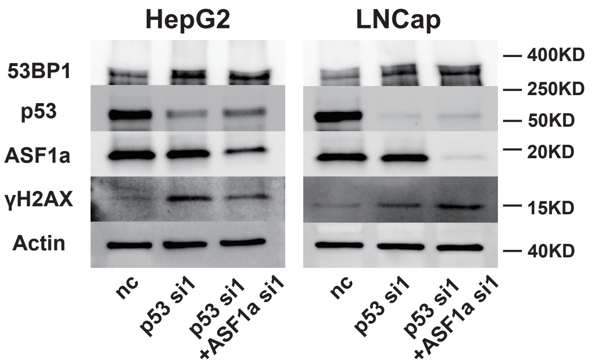

	HepG2			LNCap		
	nc	p53 si1	p53 si1 + ASF1a si1	nc	p53 si1	p53 si1 + ASF1a si1
53BP1	1.00	1.82	1.81	1.00	2.42	2.59
p53	1.00	0.43	0.62	1.00	0.08	0.11
ASF1a	1.00	0.87	0.37	1.00	0.87	0.07
γH2AX	1.00	3.54	2.42	1.00	2.09	2.20

B

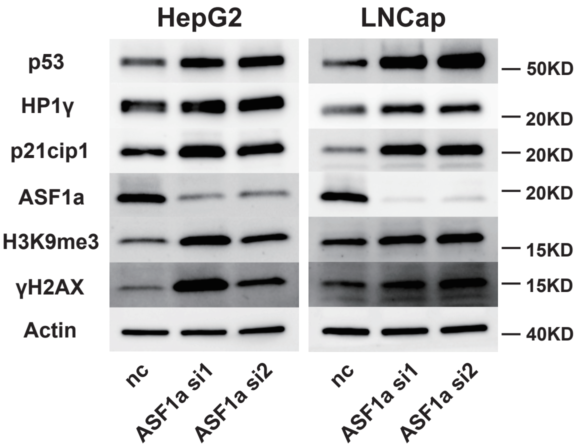

	HepG2			LNCap		
	nc	ASF1a si1	ASF1a si2	nc	ASF1a si1	ASF1a si2
p53	1,00	2,08	2,23	1,00	2,47	2,74
HP1γ	1,00	1,39	1,48	1,00	1,52	1,37
p21cip1	1,00	2,16	1,82	1,00	2,91	2,84
ASF1a	1,00	0,29	0,36	1,00	0,05	0,09
H3K9me3	1,00	2,89	2,10	1,00	1,57	1,60
γH2AX	1.00	4.27	2.34	1,00	1,76	2,33

Fig. S3

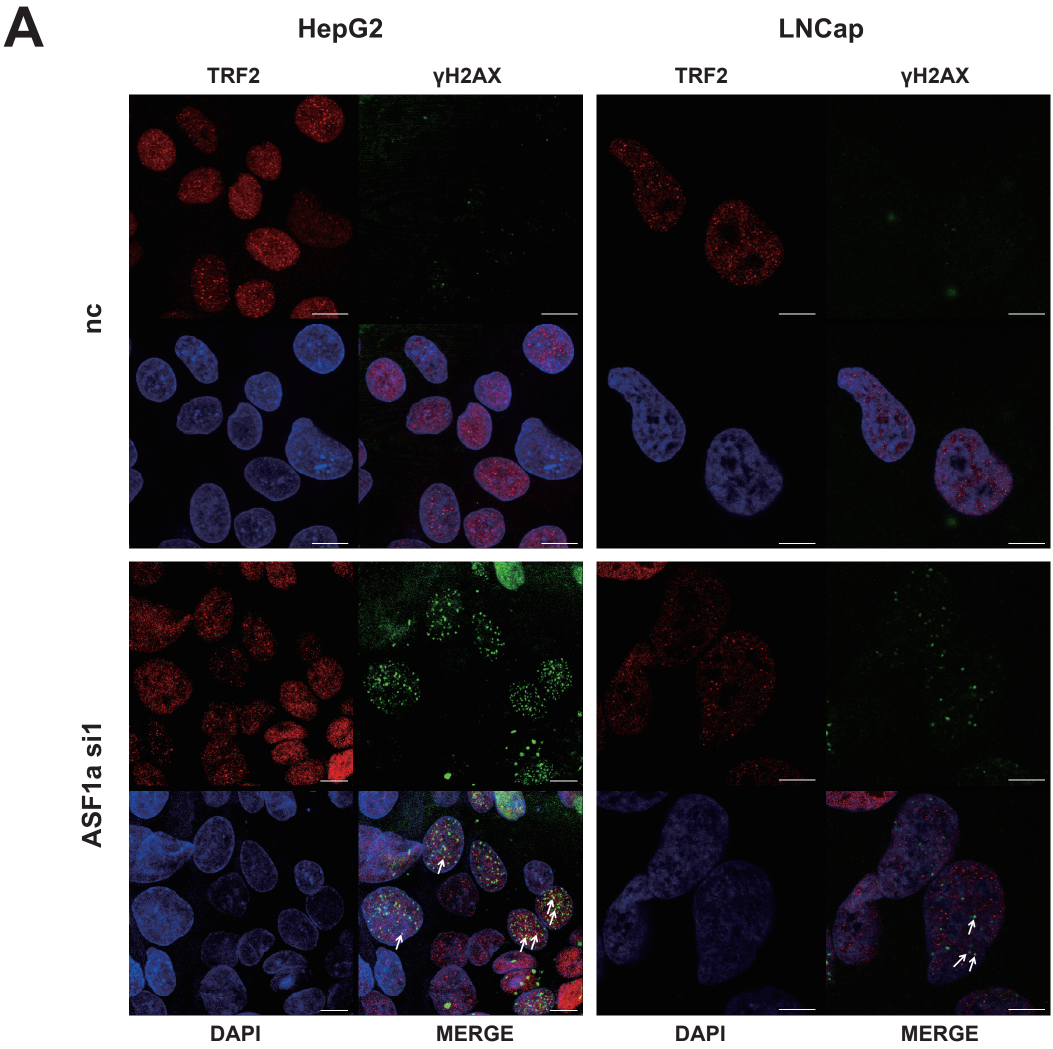
A
HepG2
LNCap
TRF2
γH2AX
TRF2
γH2AX
nc
ASF1a si1
DAPI
MERGE
DAPI
MERGE

Fig. S4

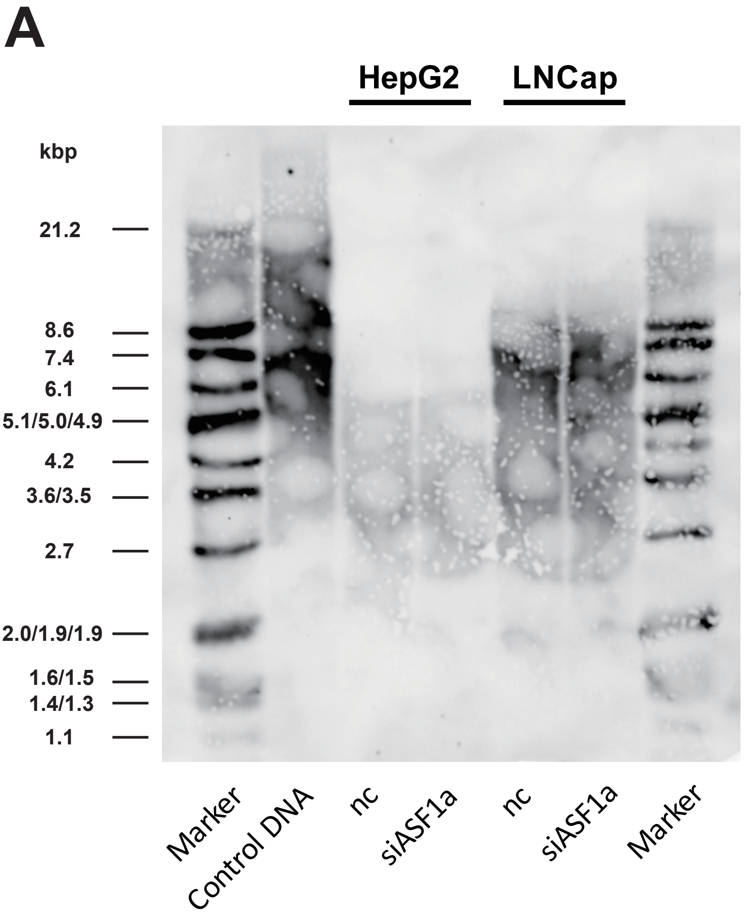
A
HepG2
LNCap
kbp
21.2
8.6
7.4
6.1
5.1/5.0/4.9
4.2
3.6/3.5
2.7
2.0/1.9/1.9
1.6/1.5
1.4/1.3
1.1
Marker
Control DNA
nc
siASF1a
nc
siASF1a
Marker

Fig. S5

A

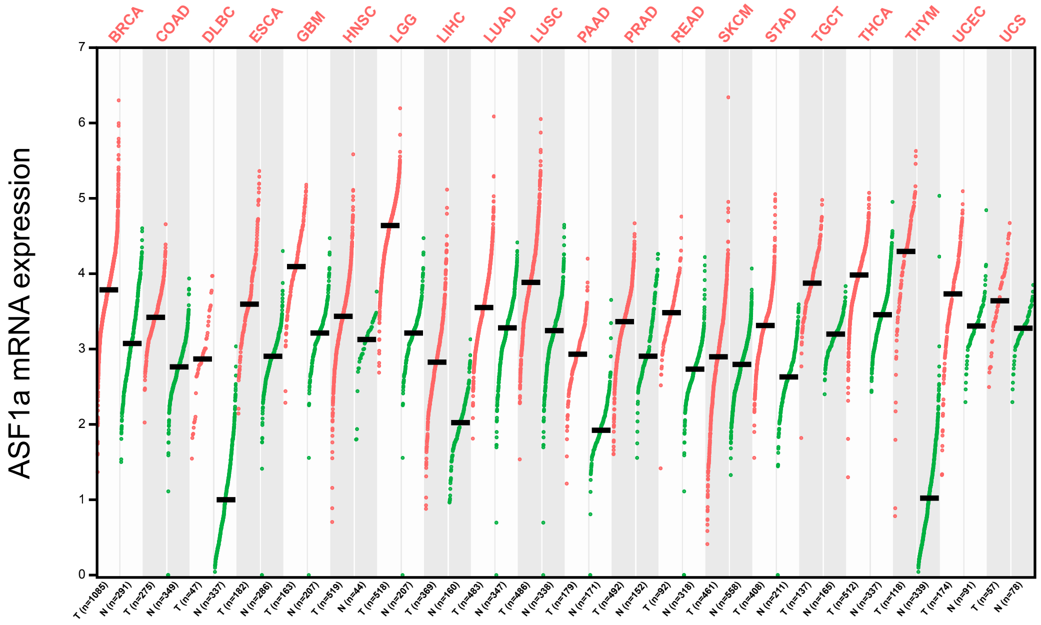

BRCA
COAD
DLBC
ESCA
GBM
HNSC
LGG
LIHC
LUAD
LUSC
PAAD
PRAD
READ
SKCM
STAD
TGCT
THCA
THYM
UCEC
UCS
ASF1a mRNA expression
0
1
2
3
4
5
6
7
T (n=1085)
N (n=291)
T (n=275)
N (n=349)
T (n=47)
N (n=337)
T (n=182)
N (n=286)
T (n=163)
N (n=207)
T (n=519)
N (n=44)
T (n=518)
N (n=207)
T (n=369)
N (n=160)
T (n=483)
N (n=347)
T (n=486)
N (n=338)
T (n=179)
N (n=171)
T (n=492)
N (n=152)
T (n=92)
N (n=318)
T (n=461)
N (n=558)
T (n=408)
N (n=211)
T (n=137)
N (n=165)
T (n=512)
N (n=337)
T (n=118)
N (n=339)
T (n=174)
N (n=91)
T (n=57)
N (n=78)

Fig. S6

Table S1. Primers and siRNA sequences used in the present study

Genes	Primers and siRNA sequences
	Primers for qPCR
ASF1a	Forward: 5’-CAGATGCAGATGCAGTAGGC-3’<br>Reversed: 5’-CCTGGGATTAGATGCCAAAA-3’
P21cip1	Forward: 5’-GCGACTGTGATGCGCTAAT-3’<br>Reversed: 5’-TAGGGCTTCCTCTTGGAGAA-3’
P16ink4a	Forward: 5’-TTCCTGGACACGCTGGT-3’<br>Reversed: 5’-CAATCGGGGATGTCTGAG-3’
P27kip1	Forward: 5’-ATGTCAAACGTGCGAGTGTCTAA-3’<br>Reversed: 5’-TTACGTTTGACGTCTTCTGAGG -3’
hTERT	Forward: 5’-CGGAAGAGTGTCTGGAGCAA-3’<br>Reversed: 5’- GGATGAAGCGGAGTCTGGA-3’
β2-M	Forward: 5’-GAATTGCTATGTGTCTGGGT-3’<br>Reversed: 5’-CATCTTCAAACCTCCATGATG-3’
	siRNAs
Negative control (nc)	5’- UCAACAUCAUCGAUCGAGAUGUAGG-3’
ASF1a (si1):	5’-UGACUGUAGAUUUGGAUUACUGCUC-3’
ASF1a (si2):	5’-UAUACUGAGACAGAAUUAAGGGAAA-3’
siP53:	5’-AACCUCUUGGUGAACCUUAGUACCT-3’
siP21cip1	5’-UGAGCCGCGACUGUGAUGCGCUAAU-3’
